# Transmission Dynamics of Large Coronavirus Disease Outbreak in Homeless Shelter, Chicago, Illinois, USA, 2020

**DOI:** 10.3201/eid2801.210780

**Published:** 2022-01

**Authors:** Yi-Shin Chang, Stockton Mayer, Elizabeth S. Davis, Evelyn Figueroa, Paul Leo, Patricia W. Finn, David L. Perkins

**Affiliations:** University of Illinois at Chicago, Chicago, Illinois, USA (Y.-S. Chang, S. Mayer, E. Figueroa, P. Leo, P.W. Finn, D.L. Perkins);; Rush University Medical Center, Chicago (E.S. Davis)

**Keywords:** COVID-19, coronavirus disease, SARS-CoV-2, severe acute respiratory syndrome coronavirus 2, viruses, respiratory infections, zoonoses, transmission dynamic, SEIR model, homeless shelter, congregate setting, reproductive number, Chicago, Illinois, United States

## Abstract

Severe acute respiratory syndrome coronavirus 2 (SARS-CoV-2) has the potential for rapid transmission in congregate settings. We describe the multidisciplinary response to an outbreak of coronavirus disease (COVID-19) in a large homeless shelter in Chicago, Illinois, USA. The response to the outbreak included 4 rounds of mass PCR testing of all staff and residents and subsequent isolation of persons who tested positive for SARS-CoV-2. We further describe the dynamics of the shelter outbreak by fitting a modified susceptible-exposed-infectious-recovered compartmental model incorporating the widespread SARS-CoV-2 testing and isolation measures implemented in this shelter. Our model demonstrates that rapid transmission of COVID-19 in the shelter occurred before the outbreak was detected; rates of transmission declined after widespread testing and isolation measures were put in place. Overall, we demonstrate the feasibility of mass PCR testing and isolation in congregate settings and suggest the necessity of prompt response to suspected COVID-19 outbreaks in homeless shelters.

The coronavirus disease (COVID-19) pandemic, caused by severe acute respiratory syndrome coronavirus 2 (SARS-CoV-2), has disproportionately affected persons living in congregate settings, including homeless shelters (*1*,*2*). People experiencing homelessness are at increased risk for SARS-CoV-2 infection because of shared living spaces and difficulty maintaining physical distance and are at increased risk for severe COVID-19 because of the high prevalence of underlying medical conditions (*3*,*4*).

Previous studies of COVID-19 in homeless shelters have reported testing results from 1 or 2 cross-sectional time points of an outbreak (*1*,*2*), but data are limited regarding the dynamics of SARS-CoV-2 transmission in homeless shelters. Community transmission was documented in Chicago, Illinois, USA, in early March (*5*), and a statewide stay-at-home order was implemented on March 14, 2020. During March–May 2020, many homeless shelters in Chicago experienced COVID-19 outbreaks (*4*). We describe an outbreak of COVID-19 in Chicago’s largest homeless shelter, including the results of repeated rounds of SARS-CoV-2 reverse transcription PCR (RT-PCR) testing. On the basis of these data, we developed a compartmental mathematical model to characterize the extent and temporal dynamics of SARS-CoV-2 infection within this shelter.

## Methods

### Study Population and Setting

Pacific Garden Mission (PGM) in Chicago is the largest homeless shelter in the midwestern United States, having a capacity for 950 residents. Most residents (referred to as overnight residents) sleep at night in large, gender-separated dormitories capable of accommodating <200 residents. During the day, these residents leave the shelter or stay collectively in large gender-separated day rooms before returning to sleep in the same dormitories but with changed bed allocations. Before the statewide stay-at-home order, the maximum length of stay for residents was 30 days. A smaller number of residents (referred to as program residents) sleep at night in smaller dormitories (ranging from 4 to 20 beds) and spend their days in the dormitories, day room, accessing services, or outside the shelter; these residents can stay in the shelter for up to 2 years depending on the services they are accessing. When the stay-at-home order was mandated, >50 residents and staff left PGM. After the statewide stay-at-home order, no residents were permitted to leave or return to the shelter, except for a select few in essential roles (e.g., employment in critical infrastructure). A total of 445 residents and staff remained at PGM.

### Origin of the Outbreak at PGM

On March 14, 2020, COVID-19 was diagnosed in a female overnight resident in her 40s at an acute-care hospital. A total of 9 other PGM residents subsequently became symptomatic and sought clinical care in March; SARS-CoV-2 infection was confirmed in 10 persons by March 31. Of these, 7 were male overnight residents, 2 were female overnight residents, and 1 was a male staff member.

### Clinical and Public Health Investigation and Response

**Figure 1:**
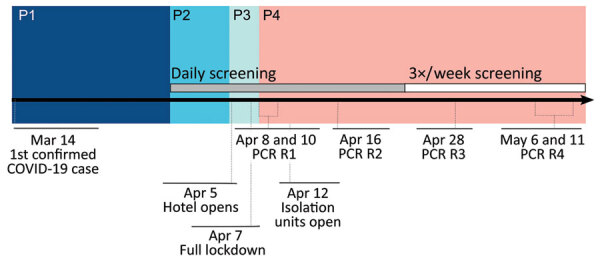
Summary timeline of COVID-19 outbreak and response at Pacific Garden Mission, a homeless shelter in Chicago, Illinois, USA, 2020. P1, prescreening (March 14–March 30); P2, symptom screening (March 30–April 5) and temporary isolation; P3, hotel opening with continued symptom screening (April 5–8); P4, mass RT-PCR testing rounds and isolation units (April 8–May 11). COVID-19, coronavirus disease; P, phase; RT-PCR, reverse transcription PCR.

For the purposes of this analysis, the investigation and response are divided into 4 phases (Figure 1). In phase 1, during March 1–29, 2020, no routine symptom screening or SARS-CoV-2 testing was conducted at PGM. Residents who sought care from staff after experiencing COVID-19–related symptoms were taken to nearby acute-care hospitals for diagnostic testing and clinical care.

In phase 2, during March 30–April 4, 2020, infection control measures were enhanced, including cleaning of frequently touched surfaces, improving the availability of hand hygiene products (e.g., alcohol-based hand sanitizer), implementing physical distancing policies, and providing facemasks to all residents (sufficient masks for universal masking were obtained by April 2). In addition, daily temperature checks and symptom screens were introduced. Residents with possible COVID-19 symptoms (persons under investigation [PUIs]) were isolated onsite. Consistent with the Centers for Disease Control and Prevention (CDC) definition at the time, residents were determined to be PUIs if they had a measured fever of >37.8C or reported a subjective fever, dry cough, shortness of breath, myalgia, sore throat, headache, fatigue, or close contact with a person who had confirmed SARS-CoV-2 infection.

In phase 3, during April 5–7, 2020, PUIs were transferred for offsite isolation at a hotel with individual rooms. Newly symptomatic residents were transferred to the hotel, on average, 1 day after reporting symptoms and were isolated onsite in the interim. Simultaneously, residents at high risk for severe disease (because of age or underlying medical conditions, as determined by an onsite physician) were also transferred offsite for protective housing in individual hotel rooms. A stricter shelter-in-place was instigated on April 7, 2020; after this date, residents were strongly discouraged from leaving, and residents who left for any reason were not permitted to return.

Phase 4 was characterized by recurrent rounds of widespread testing for SARS-CoV-2. During April 8–10, 2020, healthcare workers from local academic healthcare centers collected oronasopharyngeal swab specimens from all consenting staff and residents. Testing was offered to all residents and staff who had not previously tested positive for SARS-CoV-2. Specimens were tested for SARS-CoV-2 by RT-PCR, and associated clinical and epidemiologic information was collected by using a standardized questionnaire as previously described (*4*). On average, test results were returned 48 hours after specimen collection. Isolation units, staffed by clinicians 24 hours a day and with capacity for 160 persons, were established onsite for residents who tested positive for SARS-CoV-2. Isolation units were equipped with a personal protective equipment (PPE) station for medical personnel; staff and residents were regularly trained in PPE use, and the PPE station was regularly stocked with surgical and N95 masks, gloves, and gowns. Further rounds of widespread testing were conducted on April 18, April 28, and May 6. After each round, residents were isolated as described previously. Residents who became symptomatic between rounds of testing but did not have a RT-PCR–confirmed diagnosis continued to be transferred to the hotel.

### Modeling Transmission Dynamics of COVID-19 at PGM

To characterize transmission dynamics, we adapted a classic propagation dynamics compartmental model, susceptible-exposed-infectious-recovered (SEIR), to incorporate isolation and mass testing measures (Appendix). The SEIR model classifies persons in a population into 4 compartments of susceptible, exposed), infected), and recovered (or removed) and applies well to the relatively closed system of a homeless shelter, particularly after the stay-at-home order and the subsequent, stricter shelter-in-place policy. Rate of transmission is governed by 3 parameters: rate of transmission between susceptible and infectious persons (β = [*n*_contacts/infectious individual/d_ × probability_transmission given contact_]), the rate of conversion from exposed to infectious (σ = 1/*t*_incubation_, *t*_incubation_ = incubation period), and the rate of recovery (ϒ = 1/*t*_infectious_, *t*_infectious_ *= duration of infectiousness*). A system of ordinary differential equations determines the temporal progression of persons within each compartment.

**Figure 2:**
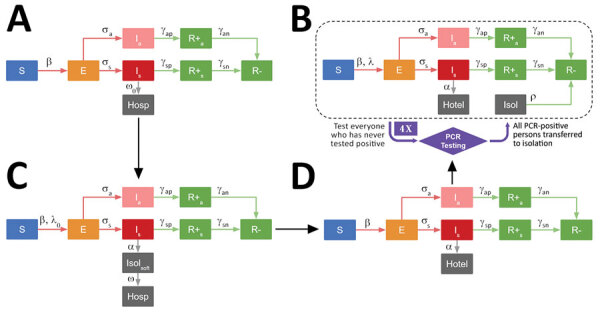
Sequential compartmental models corresponding to the 4 phases of the coronavirus disease outbreak response at Pacific Garden Mission, a homeless shelter in Chicago, Illinois, USA, 2020. A) Phase 1: prescreening (March 14–March 30); B) phase 2: symptom screening (March 30 – April 5) and temporary isolation; C) phase 3: hotel opening with continued symptom screening (April 5–8); D) phase 4: mass reverse transcription PCR testing rounds and isolation units (April 8–May 11). Corresponding description of compartments, systems of ordinary differential equations, and parameter descriptions are described in detail in the Appendix. E, Exposed; hosp, hospital; I, infectious; isol, isolation; R, recovered; S, susceptible

We adapted the SEIR compartmental model to understand the dynamics of the PGM outbreak and constructed a model consisting of 4 separate systems of ordinary differential equations corresponding to the 4 phases of outbreak response at PGM (Figure 2). In each of these phases, we altered the corresponding model parameters and compartments to represent relevant screening, testing, and isolation measures. Our model introduces a compartment for isolation units in phase 4 and a compartment for isolation dorms (before the set-up of fully staffed, PPE-stocked isolation units) in phases 2 and 3. Finally, the model includes compartments for persons who were removed to the hotel or a hospital.

Model variables including β, incubation period, infectious duration, RT-PCR–positive duration, asymptomatic percentage, and RT-PCR sensitivity were fit to early testing data (March 14–April 7, 2020) from symptomatic persons who sought care at the hospital, number of persons admitted to the hospital, number of persons moved to the hotel, and results of the 4 rounds of mass testing by using the limited memory Broyden–Fletcher–Goldfarb–Shanno (L-BFGS) optimization algorithm in R (with native R function optim) (*6*,*7*). We derived ranges of values for each optimized variable from the literature (Table 1; Appendix)**.** Basic reproduction number (R_0_), which is calculated as β/ϒ in a basic SEIR model, was calculated as β_0_/[ϒ_ap_ × [% asymptomatic] + [ϒ_sp_ × (% symptomatic)], where ϒ_ap_ is the inverse of infectious duration for asymptomatic persons and ϒ_sp_ is the inverse of infectious duration for symptomatic persons. The number of persons in different compartments at various timepoints and model parameters (representing transmission dynamics) were estimated from the fitted model (Appendix Table).

**Table 1:** Model parameters for fitting in study of transmission dynamics of coronavirus disease outbreak in homeless shelter, Chicago, Illinois, USA, 2020*

Fitted variables	Range of values fitted	Description of variable	Referenced ranges for fitting	Directly dependent model parameters	Dependent model phases	Dependent model compartments
β_0_	0–445	Initial β	NA	β, R_0_	1, 2, 3, 4	S, E
β_f__pct_β_0_	0–1	Final β as percentage of β_0_	NA	β	1, 2, 3, 4	S, E
k	0.01–2	Rate of transformation of β	NA	β	1, 2, 3, 4	S, E
t_Trans_	1–50	Day where β reaches halfway between β_0_ and β_f_	NA	β	1, 2, 3, 4	S, E
Incubation period	2.8–4.0	Time between E and I compartments	(*8*)	σ	1, 2, 3, 4	E, Ia, Is
Asymptomatic percentage	0.18–0.87	Asymptomatic percentage	(*9**,**10*)	σ_s_, σ_a_, R_0_	1, 2, 3, 4	E, Ia, Is
Infectious period for symptomatic persons, d	3–8	Infectious duration for symptomatic persons	(*11**,**12*)	ϒ_sp_, R_0_	1, 2, 3, 4	Is, R+_s_
Infectious period for asymptomatic persons, d	3–8	Infectious duration for asymptomatic persons	(*11**,**12*)	ϒ_ap_, R_0_	1, 2, 3, 4	Is, R+_a_
Period of RT-PCR–positivity for symptomatic persons, d	16–35	Duration of RT-PCR**–**positivity of symptomatic persons	(*13**,**14*)	ϒ_sn_	1, 2, 3, 4	R+_s_, R–_s_
Period of RT-PCR–positivity for asymptomatic persons, d	3–35	Duration of RT-PCR**–**positivity for asymptomatic persons	(*13**,**14*)	ϒ_an_	1, 2, 3, 4	R+_a_, R–_a_
α	0.01–1	Rate of detection of symptomatic infectious persons through screening	NA	α	2, 3, 4	Is, Isol_soft_, Hotel
λ_0__pct_β	0–1	Rate of transmission between persons in Isol_soft_ and S compartment, as a percentage of β	NA	λ_0_	2, 3	S, E
λ_isol__pct_β	0–0.5	Rate of transmission between persons in isolation units and S compartment, as a percentage of β	NA	λ_isol_	4	S, E
Isolation duration, d	14	Rate of return from isolation units to R compartment = 1/[14 d]†	NA	ρ	2, 3, 4	Isol, R
RT-PCR sensitivity	0.72–0.90	RT-PCR sensitivity	N.S. Padhye, unpub. data‡	–	1 and 2,§ 4	-
ω_0_	0.05–1.0	Rate of hospital admission of Infectious symptomatic persons before screening	NA	ω_0_	1	Is, Hosp
ω	0.05–1.0	Rate of hospital admission of Isol_soft_ symptomatic persons during phase 2	NA	ω	2	Isol_soft_, Hosp

*Details of optimization and calculation can be found in the Appendix (https://wwwnc.cdc.gov/EID/article/28/1/21-0780-App1.pdf). E, exposed; Ia, infectious asymptomatic; Is, infectious symptomatic; NA, not applicable; R+, recovered PCT-positive; R–, recovered PCR-negative; RT-PCR, reverse transcription PCR; R0, basic reproduction number; S, susceptible.

†Value not fitted.

‡https://www.medrxiv.org/content/10.1101/2020.04.24.20078949v2.

§Fitting based on hospital-based test results.

## Results

Demographic and health information of residents and staff members at PGM who had an RT-PCR test performed any time during March 14–May 11, 2020, were self-reported (Table 2). The demographic distribution of PGM residents is similar to that of a broader survey of persons experiencing homelessness in Chicago (*4*); most are men (255/358, 71%) and non-Hispanic Black (219/344, 64%), and the median age is 56 years (interquartile range 45–61 years).

**Table 2:** Self-reported characteristics of Pacific Garden Mission staff and residents, Chicago, Illinois, USA, 2020

Characteristic	No. (%)
All	429 (100)
Role	
Resident	362 (83)
Staff member	67 (17)
Age group, y	
20–29	22 (5)
30–39	40 (10)
40–49	81 (20)
50–59	144 (35)
60–69	109 (26)
>70	18 (4)
Sex	
M	301 (70)
F	131 (30)
Other	1 (0)
Race and ethnicity	
Non-Hispanic Black	266 (62)
Non-Hispanic White	92 (21)
Hispanic	48 (13)
Non-Hispanic Other	22 (5)
Smoking status	
Current smoker	133 (33)
Former smoker	112 (28)
Nonsmoker	156 (39)
Medical history	
Cardiovascular disease	85 (22)
Chronic lung disease	53 (13)
Diabetes mellitus	54 (14)
Neurologic disease	20 (5)
Chronic kidney disease	13 (3)
Immunocompromised	10 (3)
Chronic liver disease	7 (2)

**Figure 3:**
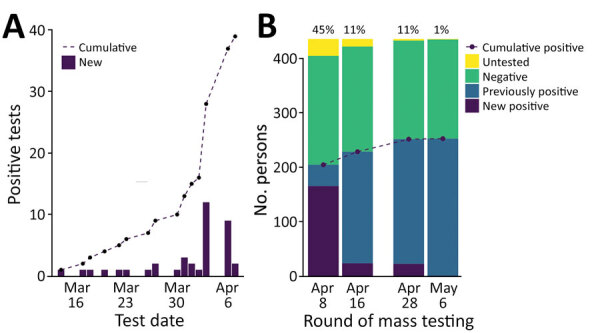
Coronavirus disease cases confirmed through reverse transcription PCR (RT-PCR) over time at Pacific Garden Mission, a homeless shelter in Chicago, Illinois, USA, 2020. A) Hospital-based positive tests before mass testing (March 14–April 7, 2020). Number of positive hospital-based RT-PCR tests per day (bars) and cumulatively (dashed line) are displayed for the period before mass testing. B) Results from each of 4 rounds of mass testing. Number of persons who were previously positive (and therefore not tested), newly positive, negative, and not tested for each round of mass RT-PCR testing are displayed; percentage of tests returning positive (n_positive_/n_tested_) are displayed above. During mass testing, 166 positive cases were detected in the first round, 24 positive cases were detected in the second round, 23 positive cases were detected in the third round, and 1 positive case was detected in the fourth round.

During phases 1, 2, and 3, SARS-CoV-2 infection was confirmed in a total of 39 persons (35 residents and 4 staff members) (Figure 3, panel A). Of those 39 positive cases, 26 were confirmed after universal symptom screening was begun in the final week before mass testing.

The first round of widespread RT-PCR testing identified 166 (45%) of 366 persons who were confirmed to be SARS-CoV-2–positive. Subsequent rounds of testing yielded substantially lower rates of positivity: 24 (11%) of 217 in round 2 (April 16), 23 (11%) of 181 in round 3 (April 28), and 1 (0.5%) of 183 in round 4 (May 6). A small percentage of residents declined testing (or were not tested for other reasons) during each round; 8% (round 1), 6% (round 2), 1% (round 3), and 1% (round 4) of residents who were eligible for testing declined. Of the 322 residents tested during widespread testing rounds, 193 (60%) tested positive at some point. Of the 62 staff members tested, 17 (27%) tested positive (Figure 3, panel B). Of all persons who tested positive, 87% reported no symptoms at the time of testing.

**Figure 4:**
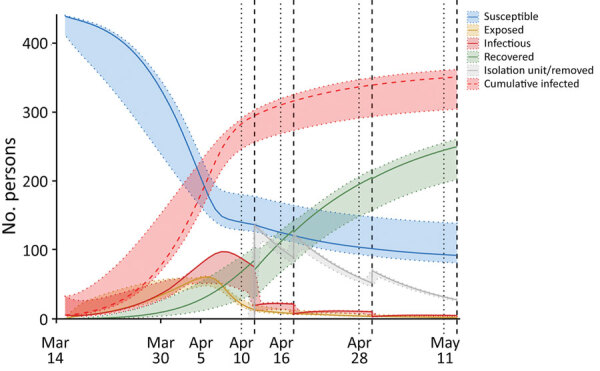
Compartmental modeling results of the coronavirus disease outbreak at Pacific Garden Mission, a homeless shelter in Chicago, Illinois, USA, 2020. The 4 phases of the outbreak are designated above the graph, and time points corresponding to each of the 4 rounds of mass testing and isolation are indicated by vertical dotted lines and vertical dashed lines. The susceptible compartment corresponds to persons who are estimated to have never been infected; exposed persons have been infected but are not yet infectious; infectious includes persons in both I_s_ and I_a_; recovered include the R+_s_, R+_a_, and R– compartments; isolation unit/removed persons tested positive by reverse transcription PCR and either left the shelter or were moved to isolation units. The discontinuities in the isolation unit/removed, infectious, and recovered curves at each of the isolation time points (dotted lines) represent persons who tested positive by reverse transcription PCR (those in the I_s_, I_a_, R+_s_, and R+_a_ compartments) at the respective testing time point (dashed lines) being moved to the Isolation Unit compartment with each of the 4 rounds of mass testing. The 95% CIs for the compartments represent maximum and minimum values for each trajectory when reperforming model optimization with β_0_ (initial transmission rate) fixed over its 95% CI (0.45–0.74) derived from initial model optimization (β_0_ = 0.60). Corresponding description of compartments, systems of ordinary differential equations, and parameter descriptions are described in detail in the Appendix.

Compartmental model trajectories are displayed for susceptible, exposed, infectious, recovered, and cumulatively infected persons over time (Figure 4). The 95% CIs of the trajectories are displayed based on model optimization across the 95% CI of initial transmission rate (β_0_ = 0.60 [95% CI 0.45–0.74]). These results demonstrate widespread transmission in the early stages of the outbreak (phases 1–3); most cases were undetected before shelterwide testing, even after the implementation of screening measures in phase 2 (Appendix Figure 1). These results suggest that ≈350 persons were cumulatively infected, compared with the 253 cases detected by RT-PCR during the outbreak. This discrepancy is driven predominantly by persons who were infected (but whose illness was undetected) early in the outbreak who stopped shedding virus before mass testing. Model fitting yielded a R_0_ of 4.5 (95% CI 2.7–4.8) (Appendix). Dependent model parameters are included (Table 3).

**Table 3:** Fitted model parameter values in study of transmission dynamics of coronavirus disease outbreak in homeless shelter, Chicago, Illinois, USA, 2020*

Parameter	Fitted value	Description of parameter
β_0_	0.60	Initial β
β_f__pct_β_0_	0.11	Final β as percentage of β_0_
k	2.0	Rate of transformation of β
t_Trans_	23	Day where β reaches halfway between β_0_ and β_f_
σ_s_	0.098	Rate of transition from E to Is compartment = 1/(incubation period) × (% symptomatic)
σ_a_	0.26	Rate of transition from E to Ia compartment = 1/(incubation period) × (% asymptomatic)
Asymptomatic percentage	0.73	Asymptomatic percentage
ϒ_sp_	0.15	Rate of transition from Is to Rs+ compartment = 1/(infectious period for symptomatic persons)
ϒ_ap_	0.13	Rate of transition from Ia to Ra+ compartment = 1/(infectious period for asymptomatic persons)
ϒ_sn_	0.046	Rate of transition from Rs+ to R- compartment = 1/[(duration of RT-PCR–positivity) – (infectious period)] for symptomatic persons
ϒ_an_	0.12	Rate of transition from Ra+ to R- compartment = 1/[(duration of RT-PCR–positivity) – (infectious period]) for asymptomatic persons
α	0.32	Rate of detection of I symptomatic persons through screening
λ_0__pct_β	1	Rate of transmission between persons in Isol_soft_ and Susceptible compartment, as a percentage of β
λ_isol__pct_β	1	Rate of transmission between persons in isolation units and Susceptible compartment, as a percentage of β
ρ	1/14 d*	Rate of return from isolation units to Recovered compartment = 1/[14 d]†
PCR sensitivity	0.90	RT-PCR sensitivity
ω_0_	0.75	Rate of hospital admission of Infectious symptomatic persons before screening
ω	0.39	Rate of hospital admission of Isol_soft_ symptomatic persons during phase 2

*Details of optimization and calculation can be found in the Appendix. E, exposed; I, infectious; RT-PCR, reverse transcription PCR. †Value not fitted.

## Discussion

In this study, we document a COVID-19 outbreak in a large homeless shelter involving a high number of residents; laboratory-confirmed SARS-CoV-2 infection was diagnosed in >50% of all residents and staff. Our results suggest that many others were infected before the availability of widespread testing, indicating that nearly all residents and staff were likely infected during this outbreak.

Our data represent comprehensive characterization of a COVID-19 outbreak and response in a large homeless shelter and highlight the potential for high transmission rates that could lead to rapid, exponential growth of COVID-19 outbreaks in closed, congregate settings. Our modeling results suggest that most cases were undetected before widespread testing (Figure 4; Appendix Figure 1), even after symptom screening measures began. As a result, the cumulative number of infections detected by the end of the outbreak was likely substantially underestimated. 

Our modeling results yielded an R_0_ value of 4.5, which is higher than R_0_ estimated from analyses of early community spread (R_0_ estimates 1.4–3.9) (*15*,*16*). This rate of transmission might be explained by the difficulty of social distancing in homeless shelters, as well as higher rates of medical conditions and older age that could increase susceptibility to infection. The rate of transmission is further exacerbated by the high rate of undetected infection. In this study, 87% of those with laboratory-confirmed SARS-CoV-2 infection reported no symptoms, similar to the proportion observed in other similar populations (*2*,*4*). This low reporting rate might reflect the high prevalence of background symptoms in persons experiencing homelessness that could mask COVID-19–related symptoms or could be related to distrust of healthcare providers (*17*,*18*). The consequence of this low rate of symptom reporting is a low rate of detecting of infection and transmission in the absence of shelter-wide RT-PCR testing.

These modeling data are, however, subject to limitations. Reported parameter estimates, including the duration of viral shedding, demonstrate high population variance and are not necessarily normally distributed (*19*). A study of 21 patients experiencing mild illness demonstrated repeated negative RT-PCR tests by 10 days after symptom onset (in 90% of the patients) (*20*), and another study of 56 patients with mild-to-moderate illness reported median duration of viral RNA shedding of 24 days (*14*). Furthermore, the underlying test data were limited by the availability of widespread testing; widespread testing of congregate settings was not established in Chicago until April 2020, and no widespread testing data were available to characterize phase 1 of this outbreak. Our model accounts for this early lack of testing and fits compartmental trajectories across the entire time span of the outbreak and uses known ranges for such parameters as infectious duration and RT-PCR–positive duration, but it inevitably simplifies some complexity of the context. This simplification, in addition to the large number of fitted parameters, requires cautious interpretation of fitted parameter values. Other limitations include the assumption of a closed system; although the shelter did not allow residents to enter or leave, some high-risk residents were preemptively moved to the hotel, and some residents did inevitably leave the shelter. In addition, some staff left the shelter and returned, and the model further assumes random mixing of the shelter population (outside of isolation units).

Our data reiterate the potential for high rates of SARS-CoV-2 transmission, which could result in large COVID-19 outbreaks in congregate settings, such as homeless shelters. Our data also reinforce the CDC recommendation to perform facilitywide RT-PCR testing and effective isolation in response to cases of COVID-19 in homeless shelters (https://www.cdc.gov/coronavirus/2019-ncov/community/homeless-shelters/testing.html). Isolating several hundred residents at PGM demonstrates the feasibility of establishing supported onsite isolation even within shelter settings, although offsite supported isolation centers have also been successfully used for persons experiencing homelessness (https://chhrge.org) (*21*,*22*). Establishing robust, proactive infection prevention practices, as recommended by CDC (https://www.cdc.gov/coronavirus/2019-ncov/community/homeless-shelters/plan-prepare-respond.html), and responding rapidly with a comprehensive testing and isolation protocol are crucial to keep persons residing in homeless shelters safe from COVID-19.

Appendix Additional information about the transmission dynamics of large coronavirus disease outbreak in homeless shelter, Chicago, Illinois, USA, 2020
