# Supplementary material for: Transmission Dynamics of Large Coronavirus Disease Outbreak in Homeless Shelter, Chicago, Illinois, USA, 2020
Source: Emerg Infect Dis. 2022 Jan;28(1):76–84. doi: 10.3201/eid2801.210780 (PMC8714208; doi:10.3201/eid2801.210780)
Supplement: Appendix — Additional information about the transmission dynamics of large coronavirus disease outbreak in homeless shelter, Chicago, Illinois, USA, 2020 [file 21-0780-Techapp-s1.pdf]

Article DOI: https://doi.org/10.3201/eid2801.210780

Transmission Dynamics of Large Coronavirus Disease Outbreak in Homeless Shelter, Chicago, Illinois, USA, 2020

Appendix

Supplementary Methods: Model Construction

A total of 4 sequential compartmental models were constructed corresponding to the 4 phases of coronavirus disease (COVID-19) outbreak response at the Pacific Garden Mission homeless shelter in Chicago, Illinois, USA: phase 1: prescreening (March 14–30), phase 2: symptom screening (March 30–April 5) and temporary isolation, phase 3: hotel opening and continued symptom screening (April 5–8), phase 4: mass PCR testing rounds and isolation units (April 8–May 11) (Figure 2, https://wwwnc.cdc.gov/EID/article/28/1/21-0780-F2.htm).

Because transmission rate (β) varies as a function of the number of contacts per infectious person and probability of transmission given contact, it is expected to vary over time in our model because of removal of persons from the population (primarily into isolation units) and infection control measures. β at any given time point is thus calculated by using the transition equation below:

$$\beta = \beta_0 - \frac{\beta_0 - \beta_f}{1 + e^{(t - t_{Trans})/k}},$$ where

β_0 corresponds to the initial transmission rate, $\beta_f = \beta_f\_pct\_\beta_0 \times \beta_0$ (where $\beta_f\_pct\_\beta_0$ corresponds to final transmission rate as a percentage of β_0), t_{Trans} represents the time point at which β reaches a value halfway between β_0 and β_f, and k represents the rate of transformation between initial and final β (Table 1, https://wwwnc.cdc.gov/EID/article/28/1/21-0780-T1.htm).

The phase 1 compartments are S (Susceptible), representing the number of uninfected persons; E (Exposed), representing the number of persons who have been infected but are not yet infectious; I_s (Infectious symptomatic), representing the number of persons who are infectious and symptomatic (or will become symptomatic); I_a (Infectious asymptomatic), representing

persons who are infectious and asymptomatic; $R+_s$ (Rps, Recovered symptomatic persons, PCR-positive), representing recovered symptomatic persons who are still PCR-positive; $R+_a$ (Rpa, Recovered asymptomatic persons, PCR-positive), representing recovered asymptomatic persons who are still PCR-positive; R– (Rn, Recovered, PCR-negative), representing persons who have recovered from infection; and Hospital (Hs), representing persons who tested positive through hospital-based PCR, most of whom were admitted. These are the ordinary differential equations (ODEs) for phase 1:

$$dS = -\beta \times S \times (Is+Ia)$$

$$dE = \beta \times S*(Is+Ia) - \sigma s \times E - \sigma a \times E$$

$$dIs = \sigma s \times E - \Upsilon sp \times Is - \omega 0 \times Is$$

$$dIa = \sigma a \times E - \Upsilon ap \times Ia$$

$$dRps = \Upsilon sp \times Is - \Upsilon sn \times Rps$$

$$dRpa = \Upsilon ap \times Ia - \Upsilon an \times Rpa$$

$$dRn = \Upsilon sn \times Rps + \Upsilon an \times Rpa$$

$$dHs = \omega 0 \times Is$$

β: rate of transmission between Susceptible and Infectious persons

σ_s: rate of transition from E to $I_s = 1 / t_{incubation} \times p_{symp}$, $t_{incubation}$ = incubation period, p_{symp} = percent symptomatic

σ_a: rate of transition from E to $I_a = 1 / t_{incubation} \times p_{asymp}$, $t_{incubation}$ = incubation period, p_{asymp} = percent asymptomatic

Υ_{sp}: rate of transition from I_s to $R_{ps} = 1/t_{infectious\_s}$, $t_{infectious\_s}$ = infectious period of symptomatic persons

Υ_{ap}: rate of transition from I_a to $R_{pa} = 1/t_{infectious\_a}$, $t_{infectious\_a}$ = infectious period of asymptomatic persons

Υ_{sn}: rate of transition from R_{ps} to R- = $1/[t_{pcrPos\_s} - t_{infectious\_s}]$, t_{pcrPos_s} = duration of PCR positivity for symptomatic infected persons

Υ_{an}: rate of transition from R_{pa} to R- = $1/[t_{pcrPos\_a} - t_{infectious\_a}]$, t_{pcrPos_a} = duration of PCR positivity for asymptomatic infected persons

ω_0: rate of hospital admission of I_s

(Eq. 1) ODEs for phase 1

The phase 2 compartments are similar to phase 1, but with the addition of an $Isol_{soft}$ (Isolation dorms, labeled "Q" in ODEs) compartment because of the beginning of symptom screening. These are the ODEs for phase 2:

$dS = -\beta\times S\times(Is+Ia) - \lambda0p\beta\times\beta\times S\times Q$

$dE = \beta\times S\times(Is+Ia) - \lambda0p\beta\times\beta\times S\times Q - \sigma s\times E - \sigma a\times E$

$dIs = \sigma s\times E - \Upsilon sp\times Is - \alpha\times Is$

$dIa = \sigma a\times E - \Upsilon ap\times Ia$

$dRps = \Upsilon sp\times Is - \Upsilon sn\times Rps$

$dRpa = + \Upsilon ap\times Ia - \Upsilon an\times Rpa$

$dRn = \Upsilon sn\times Rps + \Upsilon an\times Rpa$

$dQ = -\omega\times Q + \alpha\times Is$

$dHs = \omega\times Q$

β: rate of transmission between Susceptible and Infectious persons

$\lambda0p\beta$: rate of transmission between Susceptible and $Isol_{soft}$(Q) persons, as a percentage of β

σ_s: rate of transition from E to I_s = $1 / t_{incubation} \times p_{symp}$, $t_{incubation}$ = incubation period, p_{symp} = percent symptomatic

σ_a: rate of transition from E to I_a = $1 / t_{incubation} \times p_{asymp}$, $t_{incubation}$ = incubation period, p_{asymp} = percent asymptomatic

Υ_{sp}: rate of transition from I_s to R_{ps} = $1/t_{infectious\_s}$, $t_{infectious\_s}$ = infectious period of symptomatic persons

Υ_{ap}: rate of transition from I_a to $R_{pa} = 1/t_{infectious\_a}$, $t_{infectious\_a}$ = infectious period of asymptomatic persons

Υ_{sn}: rate of transition from R_{ps} to $R_n = 1/[t_{pcrPos\_s} - t_{infectious\_s}]$, t_{pcrPos_s} = duration of PCR-positivity for symptomatic infected persons

Υ_{an}: rate of transition from R_{pa} to $R_n = 1/[t_{pcrPos\_a} - t_{infectious\_a}]$, t_{pcrPos_a} = duration of PCR-positivity for asymptomatic infected persons

α: rate of transition from I_s to Q

ω: rate of transition from Q to Hosp

(Eq. 2) ODEs for phase 2

The phase 3 compartments are similar to phase 2, but with the replacement of the Hospital (Hosp) and $Isol_{soft}$(Q) compartments with Hotel (Ht) because of the opening of a hotel for homeless persons suspected to have COVID-19. All symptomatic PCR-positive persons were moved to the hotel upon a positive test. These are the ODEs for phase 3:

$dS = -\beta \times S \times (Is+Ia)$

$dE = \beta \times S \times (Is+Ia) - \sigma s \times E - \sigma a \times E$

$dIs = \sigma s \times E - \Upsilon sp \times Is - \alpha \times Is$

$dIa = \sigma a \times E - \Upsilon ap \times Ia$

$dRps = \Upsilon sp \times Is - \Upsilon sn \times Rps$

$dRpa = + \Upsilon ap \times Ia - \Upsilon an \times Rpa$

$dRn = \Upsilon sn \times Rps + \Upsilon an \times Rpa$

$dHt = + \alpha \times Is$

β: rate of transmission between Susceptible and Infectious persons

σ_s: rate of transition from E to $I_s = 1 / t_{incubation} \times p_{symp}$, $t_{incubation}$ = incubation period, p_{symp} = percent symptomatic

σ_a: rate of transition from E to $I_a = 1 / t_{incubation} \times p_{asymp}$, $t_{incubation}$ = incubation period, p_{asymp} = percent asymptomatic

Υ_{sp}: rate of transition from I_s to R_{ps} = $1/t_{infectious\_s}$, $t_{infectious\_s}$ = infectious period of symptomatic persons

Υ_{ap}: rate of transition from I_a to R_{pa} = $1/t_{infectious\_a}$, $t_{infectious\_a}$ = infectious period of asymptomatic persons

Υ_{sn}: rate of transition from R_{ps} to R_n = $1/[t_{pcrPos\_s} - t_{infectious\_s}]$, t_{pcrPos_s} = duration of PCR positivity for symptomatic infected persons

Υ_{an}: rate of transition from R_{pa} to R_n = $1/[t_{pcrPos\_a} - t_{infectious\_a}]$, t_{pcrPos_a} = duration of PCR positivity for asymptomatic infected persons

α: rate of transition from I_s to Hotel

(Eq. 3) ODEs for phase 3

The phase 4 compartments are similar to phase 3, but with the addition of the Isol compartment because of the implementation of Isolation Units for persons who tested positive during mass PCR screens. At each of the 4 isolation time points (2 days after each testing point), the number of persons in the I_s, I_a, R_{ps}, and R_{pa} compartments who are simulated to test positive ($\text{Sensitivity}_{PCR} \times n_{\text{individuals in each compartment on test day}}$) are moved to the Isol compartment. The phase 4 ODEs are thus propagated in 4 separate phases corresponding to the 4 testing rounds. These are the ODEs for phase 4:

$dS = -\beta \times S \times (Is+Ia) - \lambda \times S \times Isol$

$dE = \beta \times S \times (Is+Ia) - \lambda \times S \times Isol - \sigma s \times E - \sigma a \times E$

$dIs = \sigma s \times E - \Upsilon sp \times Is - \alpha \times Is$

$dIa = \sigma a \times E - \Upsilon ap \times Ia$

$dRps = \Upsilon sp \times Is - \Upsilon sn \times Rps$

$dRpa = + \Upsilon ap \times Ia - \Upsilon an \times Rpa$

$dRn = \Upsilon sn \times Rps + \Upsilon an \times Rpa$

$dHt = + \alpha \times Is$

$dIsol = - \rho \times Isol$

β: rate of transmission between Susceptible and Infectious persons

$\lambda p\beta$: rate of transmission between Susceptible and Isol persons, as a percentage of β

σ_s: rate of transition from E to I_s = 1 / $t_{incubation}$ × p_{symp}, $t_{incubation}$ = incubation period, p_{symp} = percent symptomatic

σ_a: rate of transition from E to I_a = 1 / $t_{incubation}$ × p_{asymp}, $t_{incubation}$ = incubation period, p_{asymp} = percent asymptomatic

Υ_{sp}: rate of transition from I_s to R_{ps} = $1/t_{infectious\_s}$, $t_{infectious\_s}$ = infectious period of symptomatic persons

Υ_{ap}: rate of transition from I_a to R_{pa} = $1/t_{infectious\_a}$, $t_{infectious\_a}$ = infectious period of asymptomatic persons

Υ_{sn}: rate of transition from R_{ps} to R_n = $1/[t_{pcrPos\_s}- t_{infectious\_s}]$, t_{pcrPos_s} = duration of PCR positivity for symptomatic infected persons

Υ_{an}: rate of transition from R_{pa} to R_n = $1/[t_{pcrPos\_a}- t_{infectious\_a}]$, t_{pcrPos_a} = duration of PCR positivity for asymptomatic infected persons

α: rate of transition from I_s to Hotel

ρ: rate of transition from Isol to R_n = $1/t_{isolation}$, $t_{isolation}$ = duration of isolation (14 days)

(Eq. 4) ODEs for phase 4

Supplementary Methods: Model Fitting

We constructed a function to propagate all 4 model phases sequentially, and the *optim* function within the R programming language *stats* package was used to fit model parameters with the L-BFGS optimization algorithm. The L-BFGS optimization algorithm is a quasi-Newton algorithm chosen for its stability and efficiency in handling optimization problems with large numbers of parameters. Appendix Table 1 lists the data points, values, and weights by which root mean log squared error was minimized for model fitting. Ranges of values for each optimized variable were derived from the literature. For example, reverse transcription PCR sensitivity was fit between 0.72 and 0.9 on the basis of reported ranges (*1*). Asymptomatic percentage was fit between 0.18 and 0.87 on the basis of literature estimates (*2*,*3*), as well as our results from self-reported symptoms at time of specimen collection, which showed an

asymptomatic percentage of 87%. Infectious period (separately for symptomatic and asymptomatic persons) was fit to values between 3 and 8 days on the basis of a virologic analysis assessing duration of active severe acute respiratory syndrome coronavirus 2 replication in the upper respiratory tract (*4*). Duration of PCR-positivity was fit to values from 16 to 35 days for symptomatic persons and 3–35 days for asymptomatic persons on the basis of studies with repeated PCR testing of nasopharyngeal specimens (*5*,*6*). Table 1 lists the variables that were optimized, along with the range of values supplied as boundaries to the L-BFGS optimization algorithm and the dependent model parameters. The optimization converged successfully within 500 iterations. Table 3 (https://wwwnc.cdc.gov/EID/article/28/1/21-0780-T3.htm) lists the fitted model parameter values, and Appendix Figure 2 displays the modeled data points against real data points.

Standard error of transmission rate (β_0), which is the most critical and uncertain parameter in our model, was derived as the square root of the diagonal elements of the inverse (negative) Hessian matrix evaluated at the optimum parameter values (*7*) (the Hessian matrix is returned by the *optim* function in R); the 95% CI of β_0 was thus calculated as 0.45–0.74. The Hessian derivation of CIs is only valid when the optimum parameter values are in the interior of the parameter space. This was not the case for all of our parameters, because we imposed certain narrow constraints on the basis of values reported in the literature, such as incubation period, infectious duration, and asymptomatic percentage. CIs for the basic reproduction number (R_0) were thus generated by reperforming model optimization with β_0 (initial transmission rate) fixed at equally spaced values over its 95% CI derived from initial model optimization. The R_0 CIs represent maximum and minimum values across all iterations (n = 20 equally spaced values of β_0 from 0.45–0.74) of this model optimization.

Because a bootstrapping strategy was not viable with our dataset (relatively few input data points, with certain data points of critical importance for modeling [i.e., number of positive cases from the 1st round of widespread PCR testing]), CIs for the trajectories of the different compartments were estimated in a similar manner to calculation of R_0 CIs. These intervals are represented as the maximum and minimum trajectory values across all iterations of model optimization with β_0 fixed across its 95% CI (Figure 4).

To characterize collinearity between fitted parameter values, we added noise to the input data to which our model was fitted and reperformed optimization 100 times. For each optimization, each input data point (e.g., number of PCR-positive persons during the first round of widespread PCR testing) was independently perturbed by adding a random percentage to the original data point, sampled randomly from a uniform distribution from −20% to 20%. The resultant data points used for fitting were each between 80% and 120% of their original values. We then correlated the fitted parameter values from the 100 rounds of optimization in a pairwise fashion to identify collinear parameters. Expected pairs of parameters were correlated, including an inverse correlation between symptomatic infectious period and symptomatic PCR-positive period (Pearson's correlation coefficient, $R = -0.44$); these collinearities are expected, because they contribute to the total period during which persons will test positive. However, the initial transmission rate (β_0) was only significantly correlated with symptomatic infectious period ($R = -0.23$), which was narrowly constrained in our model between 3 and 8 days. Further, the R_0 was not significantly correlated with any of the modeled parameters except for β_0.

Appendix References

1. He X, Lau EHY, Wu P, Deng X, Wang J, Hao X, et al. Author correction: Temporal dynamics in viral shedding and transmissibility of COVID-19. Nat Med. 2020;26:1491–3. PubMed https://doi.org/10.1038/s41591-020-1016-z

2. Ing AJ, Cocks C, Green JP. COVID-19: in the footsteps of Ernest Shackleton. Thorax. 2020;75:693–4. PubMed https://doi.org/10.1136/thoraxjnl-2020-215091

3. Mizumoto K, Kagaya K, Zarebski A, Chowell G. Estimating the asymptomatic proportion of coronavirus disease 2019 (COVID-19) cases on board the Diamond Princess cruise ship, Yokohama, Japan, 2020. [Erratum in: Euro Surveill. 2020;25:20200604c]. Euro Surveill. 2020;25:2000180. PubMed https://doi.org/10.2807/1560-7917.ES.2020.25.10.2000180

4. Wölfel R, Corman VM, Guggemos W, Seilmaier M, Zange S, Müller MA, et al. Virological assessment of hospitalized patients with COVID-2019. [Erratum in: Nature. 2020;588:E35.]. Nature. 2020;581:465–9. PubMed https://doi.org/10.1038/s41586-020-2196-x

5. Bullard J, Dust K, Funk D, Strong JE, Alexander D, Garnett L, et al. Predicting infectious SARS-CoV-2 from diagnostic samples. Clin Infect Dis. 2020;71:2663–6. PubMed https://doi.org/10.1093/cid/ciaa638

6. Yang Z, Zeng Z, Wang K, Wong SS, Liang W, Zanin M, et al. Modified SEIR and AI prediction of the epidemics trend of COVID-19 in China under public health interventions. J Thorac Dis. 2020;12:165–74. PubMed https://doi.org/10.21037/jtd.2020.02.64

7. Pawitan Y. In all likelihood: statistical modelling and inference using likelihood. New York: Oxford University Press; 2013.

Appendix Table. Datapoints for model fitting in study of transmission dynamics of coronavirus disease outbreak in homeless shelter, Chicago, Illinois, USA, 2020

Phase	Datapoints	Values	Weight
Phase 1	Cumulative no. hospital-based PCR positives per day during March 14–30	1–10	1 (summed across dates)
Phase 2	No. persons who tested positive by PCR during phase 2 (Apr 5)	18	1
	No. persons who were admitted to the hospital during phase 2	7	1
Phase 3	No. persons moved to hotel	26	1
Phase 4	No. positives in each of 4 rounds of mass PCR testing	166, 24, 23, 1	2 (summed across 4 rounds)
	No. persons moved to hotel between rounds of mass testing	20 (between 1 and 2), 4 (between 2 and 3), 0 (between 3 and 4)	1 (summed across 3 periods)

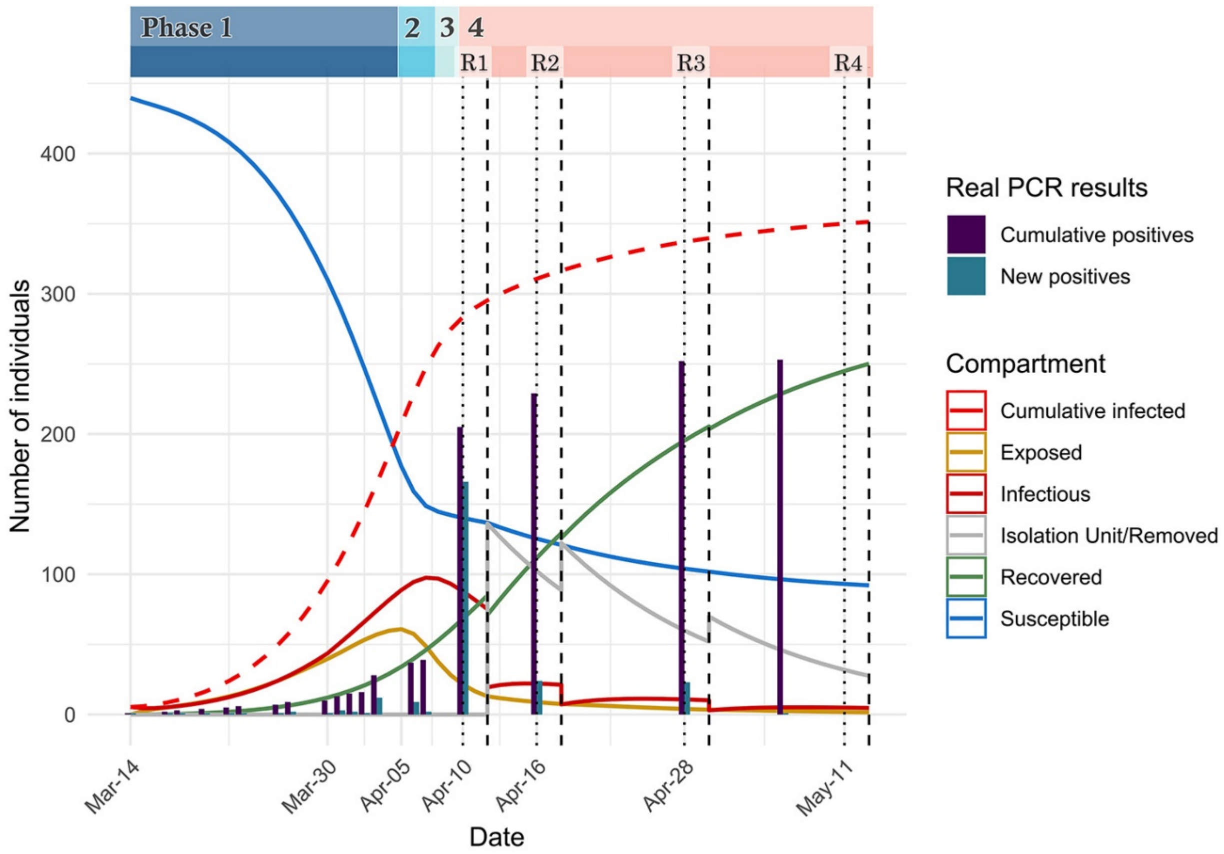

Appendix Figure 1. Compartmental modeling results superimposed with real PCR data from coronavirus disease outbreak at homeless shelter, Chicago, Illinois, USA. Compartmental modeling results (Figure 4, https://wwwnc.cdc.gov/EID/article/28/1/21-0780-F4.htm) are superimposed with real PCR testing data, including both incident and cumulative positive cases. Positive cases before phase 4 were ascertained through hospital-based PCR testing of symptomatic persons, whereas positive cases during phase 4 represent positive results from shelter-wide testing. The gap between the cumulative positive tests (real data represented by bar plots) and the modeled cumulative infections primarily represent persons who were infected (but whose illness was not detected) early in the outbreak and recovered before mass PCR testing.

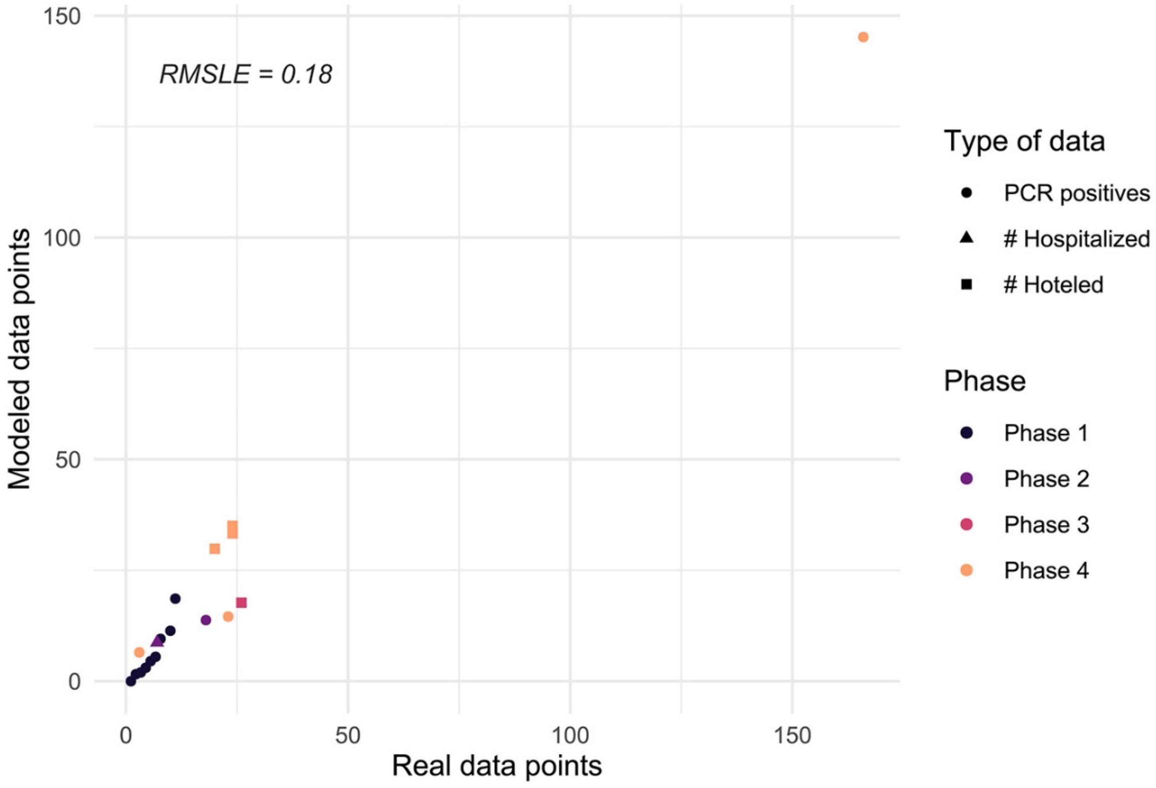

Appendix Figure 2. Modeled data points compared with real data points in study of transmission dynamics of coronavirus disease outbreak in homeless shelter, Chicago, Illinois, USA, 2020. Real data points from hospital-based PCR, shelter-wide PCR testing, and numbers of persons hospitalized or moved to the hotel are plotted against data points yielded by model fitting (Appendix Table 1). The minimized RMSLE was minimized at a value of 0.18. RMSLE, root mean square log error.
